# Effect of a Hygiene Protocol on Denture-Related Stomatitis Remission, Local Inflammatory Factors, and Hemodynamic Responses by Arterial Pressure

**DOI:** 10.3390/antibiotics11101320

**Published:** 2022-09-28

**Authors:** Adriana Barbosa Ribeiro, Aline Barbosa Ribeiro, Camila Borba de Araújo, Caroline Vieira Fortes, Lorena Mosconi Clemente, Helena de Freitas Oliveira Paranhos, Evandro Watanabe, Helio Cesar Salgado, Cláudia Helena Silva-Lovato

**Affiliations:** 1 Department of Dental Materials and Prosthesis, School of Dentistry of Ribeirão Preto, University of São Paulo, Ribeirão Preto 14040-904, Brazil; 2 Department of Physiology, Ribeirão Preto Medical School, University of São Paulo, Ribeirão Preto 14040-904, Brazil; 3 Department of Restorative Dentistry, School of Dentistry of Ribeirão Preto, University of São Paulo, Ribeirão Preto 14040-904, Brazil

**Keywords:** denture-related stomatitis, treatment, microorganisms, cytokines, saliva, arterial pressure

## Abstract

Denture-related stomatitis (DRS) is frequent oral inflammation in complete denture wearers. This study evaluated the effect of a hygiene protocol on DRS remission, local inflammatory factors, and hemodynamic responses. Thirty-three individuals were enrolled in the study. The outcomes were measured before and after 10 days of a hygiene protocol treatment consisting of brushing the palate with a soft brush and water and denture brushing with a denture-specific brush and mild soap, as well as immersion of the denture for 20 min in a 0.25% sodium hypochlorite solution. Data were analyzed by paired Wilcoxon for biofilm removal and CFU count of microorganisms. The paired T test was used to assess salivary MUC 1, cytokines, and arterial pressure (*p* < 0.05). A significant difference was found in the DRS degree (*p* < 0.001), biofilm (*p* < 0.001), microbial load of *Candida* spp. (*p* < 0.001), Gram-negative (*p* < 0.004), *Staphylococcus* spp. (*p* < 0.001), and *S. mutans* (*p* < 0.001) of the denture, and *S. mutans* (*p* < 0.001) of the palate after use of the protocol. The salivary flow (*p* = 0.2) and pH (*p* = 0.97) did not change; there was an increase of MUC 1 (*p* = 0.049) and a decrease in IL-6 (*p* = 0.038), IL-2 (*p* = 0.04), IL-10 (*p* = 0.041), and IFNγ (*p* = 0.04). There was also a decrease in systolic (*p* = 0.012) and mean arterial pressure (*p* = 0.02). The current hygiene protocol reduced the inflammation degree of DRS and promoted an improvement of local inflammatory factors and a reduction in the systolic arterial pressure of the patients.

## 1. Introduction

Edentulism, which may involve partial or total loss of tooth elements, is considered a public health problem due to its impact on general health and quality of life of individuals [1]. Of note, edentulism affects the masticatory capacity and, consequently, changes the individual’s eating habits and general health [1,2]. The literature shows that partially or completely edentulous patients can have an increased risk of hypertension, stroke [3,4,5,6], and cardiovascular diseases [5,6,7,8].

Furthermore, several lines of evidence have addressed the association between tooth loss, whether triggered by caries or periodontal disease, and abnormal levels of arterial pressure [9]. Prominent severe edentulism is considered the most serious oral condition associated with an increase of systolic arterial pressure [10].

Oral rehabilitation with complete dentures may promote a direct improvement in patients’ psychological health, aesthetics, or dietary/nutrition state [1]. However, the literature points out that the permanence of an abiotic structure in the oral cavity can favor biofilm development [11,12], thus promoting imbalance in the oral microbiota [13], and favoring the appearance of denture-related stomatitis (DRS) [11,12].

DRS is a biofilm-mediated chronic inflammation mainly rich in *Candida* spp. and bacterial strains [11,12] and can be aggravated by friction of the dentures with the mucosa, changes of the pH and salivary flow, restriction of the tongue′s action on the palate, and a reduction in mucins, such as mucin 1 (MUC 1), which are components of the physical barrier of the oral epithelium. This undesirable outcome may facilitate the breakdown of the epithelial barrier and increase the risk of microorganisms entering the bloodstream [14,15,16,17,18,19].

The mechanisms of the DRS pathogenesis are not clearly defined, but it is known that this comorbidity can trigger the activation of monocytes and T cells combined with the overproduction of cytokines, such as interleukin IL-6, interferon γ [20,21], C-reactive protein (CRP), tumor necrosis factor (TNF-α), and other pro-inflammatory cytokines [5,8].

Scientific evidence indicates that a well-established hygiene protocol can have an effect on some local factors of inflammation [11,12,22] and, consequently, could promote a hemodynamic response (arterial pressure) [15]. As an effective hygiene protocol, denture brushing and immersion in sodium hypochlorite is an alternative protocol recommended by the dental clinic since it promotes stomatitis remission in mucosal membranes and biofilm reduction on acrylic base surfaces [11,12,22], it has a low cost, and can be easily implemented. Its efficacy has been related to the solvent action of lipids and fatty acids. However, little is known about the relationship of this hygiene protocol on specific responses of the inflammation process and a hemodynamic response (arterial pressure). Therefore, this study aims to evaluate the effect of hygiene-protocol-associated palate brushing, denture brushing, and denture immersion in sodium hypochlorite on the remission of DRS, biofilm, microbial load, its effect on salivary cytokines and MUC 1, as well as the hemodynamic response. The null hypotheses tested were that the local treatment of DRS would not influence the remission of DRS, biofilm reduction, microbial load reduction, salivary cytokines, and concentration of MUC 1, as well as the hemodynamic response.

## 2. Results

A total of 33 patients were included, predominantly women with a mean age of 65.94 (±5.57) years (minimum: 54 years old and maximum: 75 years old), living with first-degree relatives, married, and retired (Table 1).

### 2.1. Denture-Related Stomatitis Remission

**Figure 1:**
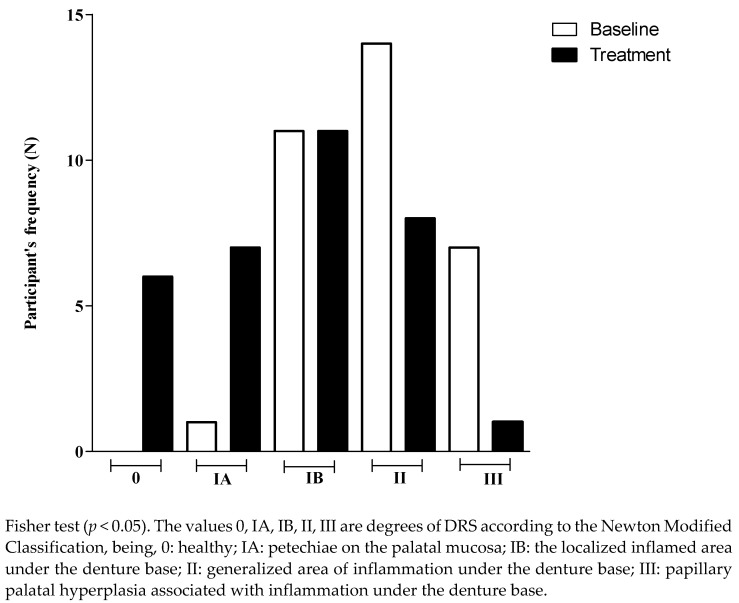
Participant’s frequency (N) according to the degree of denture-related stomatitis, at the baseline and after treatment.

Comparing the score of DRS at the baseline and after treatment with the hygiene protocol, the data show that there was significant decrease in the DRS score (Table 2). The results showed a reduction in participant frequency (n, %) with more severe degrees of DRS (Type III) and an increase in participant frequency with the lowest degree or without DRS. Figure 1 shows that those patients with more severe DRS at the baseline improved their oral conditions toward intermediate inflammation (IB or II) after treatment. Patients classified as IB at the baseline exhibited a tendency toward remission of the disease or a lower degree of DRS (0, IA) after treatment.

### 2.2. Biofilm Reduction and Antimicrobial Activity

After treatment with the hygiene protocol, there was significant reduction in the biofilm percentage of the inner surface of the upper denture when compared with the baseline (Table 3).

For dentures, the microbial load of *Candida* spp., Gram-negative bacteria, *Staphylococcus* spp., and *S. mutans* was reduced after using the hygiene protocol. For the palate, there were no changes in the CFU count of *Candida* spp., Gram-negative bacteria, as well as *Staphylococcus* spp. The hygiene protocol showed significant activity against *S. mutans*, causing a decrease in the CFU count (Table 4).

### 2.3. Salivary Parameters, Identification and Concentration of MUC 1

**Figure 2:**
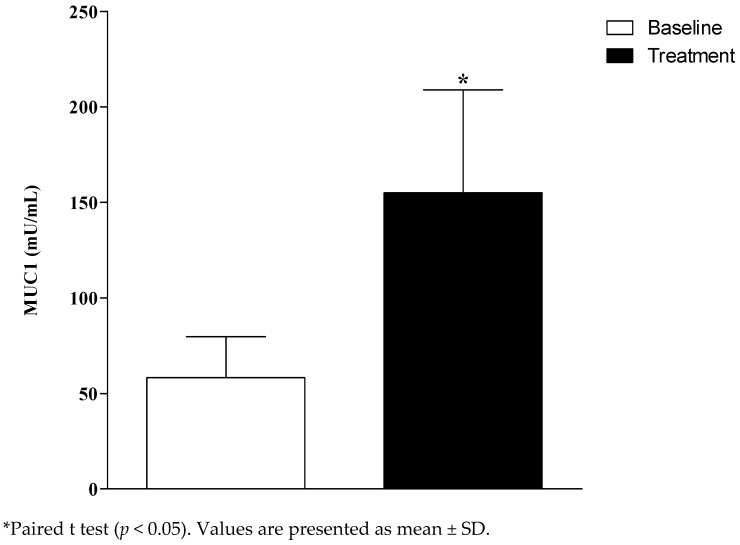
Salivary Mucin 1 (MUC 1) concentration at the baseline and after the treatment of denture-related stomatitis (DRS).

The salivary flow (SF) and pH of the unstimulated saliva of the participants were not significantly different between the baseline and after the treatment (SF baseline: 0.4; SF treatment: 0.6; *p* = 0.2; pH baseline: 7.2; pH treatment: 7.2; *p* = 0.97). There was a significant increase of salivary concentration of MUC 1 (mU/mL) after the treatment (*p* = 0.049; Figure 2).

### 2.4. Evaluation of Cytokines

**Figure 3:**
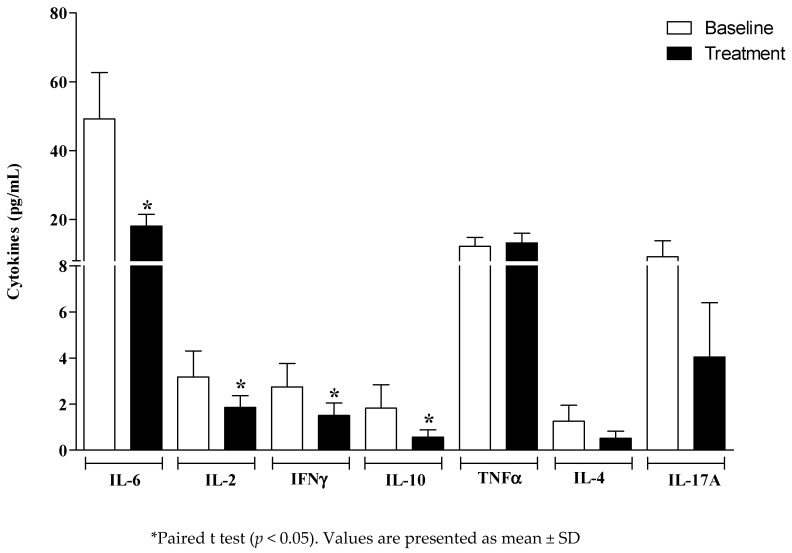
Salivary cytokines at the baseline and after the treatment of denture-related stomatitis (DRS).

After the treatment of the DRS, there were significant reductions in the salivary concentrations of IL-6 (*p* = 0.038), IL-2 (*p* = 0.04), IFNγ (*p* = 0.04), and IL-10 (*p* = 0.041), suggesting a protective anti-inflammatory effect in relation to DRS. However, no significant change was observed for TNF-α, IL-4, and IL-17A (Figure 3).

### 2.5. Evaluation of the Arterial Pressure

**Figure 4:**
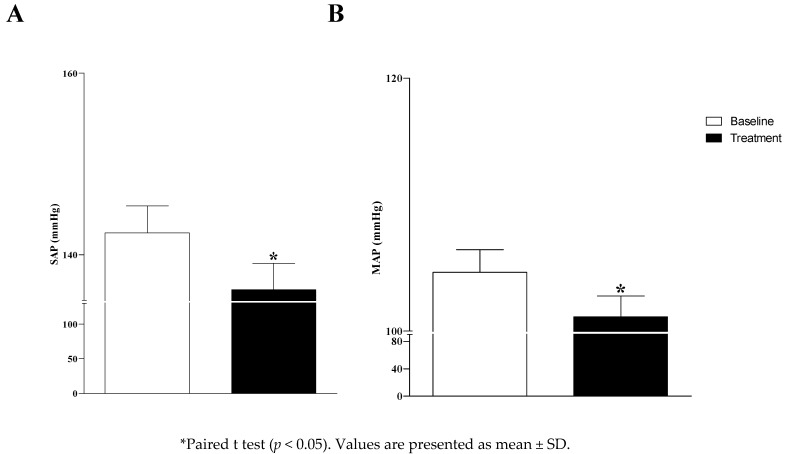
Systolic arterial pressure (SAP; panel (**A**)) and mean arterial pressure (MAP; panel (**B**)) on the baseline and after the treatment of denture-related stomatitis (DRS).

The systolic arterial pressure (SAP-baseline: 142 ± 3 vs. treatment: 136 ± 3; *p* = 0.012) and the mean arterial pressure (MAP-baseline: 105 ± 2 vs. treatment: 100 ± 1; *p* = 0.02) were reduced after the treatment of DRS (Figure 4); however, there was no effect on diastolic arterial pressure (DAP-baseline: 86 ± 2 vs. treatment: 84 ± 1; *p* = 0.36).

## 3. Discussion

Use of the hygiene protocol contributed to the remission of DRS and control of biofilm and target microorganisms; as well as this, the hygiene protocol further promoted a regulation of salivary cytokines and MUC 1. These effects may have affected the hemodynamic response, since a slight, but significant, reduction of arterial pressure was observed. Based on these results, the null hypotheses were rejected. Although other studies have evaluated the effect of hygiene protocols on stomatitis remission, biofilm control, and microbial load reduction, to the best of our knowledge, this is the first observational study that has evaluated the relationship of these variables with salivary cytokines, MUC 1 expression, and hemodynamic response evaluated by arterial pressure. The results showed that the hygiene protocol has a positive effect on all of these variables. Clinically, this can affect the prevention of local inflammation with systemic repercussions, highlighting the need for awareness of basic oral health guidelines, aiming at reducing financial costs for public health treatment.

This protocol was conducted with a completely edentulous population, the majority being women, with an average age of 65.94 years, married, living with family, and with completed high school education or less. Furthermore, 93.94% of patients had a socioeconomic status of between one to three minimum salaries, that is, almost all were from a low-income socioeconomic demographic. This sample is characteristic of individuals who wear complete dentures, similarly to other studies [11,12,23]. Less-educated subjects with dentures can have more impaired oral health than those with more education and younger subjects with dentures perceive their oral health as more impaired than older denture-wearing subjects [24]. Regarding this aspect, the demographic characteristics were limited by the small number of subjects with dentures at a younger age and with more education. Therefore, the characteristics of the sample assume the same effects for age, education, and socioeconomic status for all denture-wearing subjects.

The reduction of the biofilm, a complex polymicrobial composed of yeast, bacteria, and polysaccharides is an important result because the proliferation of microorganisms on the surfaces of the dentures stimulates a local inflammatory process. Of note, the decrease in microbial load of the *Candida* spp. is important because this microorganism is most prevalent in the biofilm of complete denture wearers and has been directly related to DRS [11,12,22]. The results of the present study reinforce this issue, since the reduced CFU count of *Candida* spp. of the denture was accompanied by a decrease in the number of individuals with a severe degree of DRS. The current hygiene protocol decreased the CFU/mL count of *Candida* spp. by more than 1 log, confirming its antimicrobial efficacy [25].

The effect of the hygiene protocol was also tested regarding the bacteria commonly found in the multi-species biofilm that interact with each other [11,12]. In general, the results showed a decrease in the microbial load of the denture after use of the hygiene protocol, while the microbial load of the palate was not influenced, with the exception of *S. mutans*. These results suggest that denture hygiene may be the most important factor for DRS control [11,12,22]. The reduction of the microbial load of bacteria is important because of the interaction between *Candida* and different bacteria [26], suggesting that the global reduction of the microbial load impacts the remission of DRS, although the literature particularly emphasizes *Candida* species.

There was a significant increase of salivary concentration of MUC 1 (mU/mL) after the use of the hygiene protocol. The relationship between mucins and bacteria is somewhat paradoxical. This is because mucins protect the oral epithelium from bacterial colonization, but they can also serve as sites of bacterial attachment [27,28]. A mechanism based on the binding of bacteria to MUC 1 on oral surfaces would initiate a signaling cascade and an increase in the production of pro-inflammatory cytokines by epithelial cells. An increased production of cytokines can lead to the upregulation of MUC 1 gene expression, resulting in the increase of the number of mucin molecules on the cell surface. This would lead to a strengthening of the protective barrier and a decrease in bacterial invasion [16]. Based on the physiology of protection and upregulation of MUC 1 gene expression, it can be assumed that in the present study, with the reduction of microbial load and remission of stomatitis, there was an increase in free MUC 1 glycoproteins (without bacterial adhesion) in saliva (extracellular medium).

Clinical and preclinical studies show a correlation between increased levels of IL-6 and cardiovascular changes, for instance, in arterial hypertension [3,4]. The results of the current study showed a reduction of the pro-inflammatory cytokine IL-6 after using the current hygiene protocol, suggesting a lower predisposition to cardiovascular alterations. Thus, the hypothesis is that the antimicrobial activity of the hygiene protocol used reduced the CFU count of microorganisms, promoting a balance among them, which is directly related to the decrease of local inflammation and the decrease of IL-6. Previous studies have identified that inflammation and a cytokine cascade appear to be the most likely common pathways correlated with DRS and cardiovascular disease [5,8,29,30].

The results also show that the salivary concentration of cytokine IL-2 was reduced, and this outcome can be justified by the association between *Candida albicans* and elevated interleukin-2 production in cultures from patients with and without denture stomatitis. *Candida* infection plays an important role in the immunomodulation of this cytokine and in the occurrence of denture stomatitis lesions [31]. With the reduction of the microbial load of *Candida* spp., the salivary concentration of IL-2 may have been reduced after using the current hygiene protocol. There was a reduction of IFNγ and IL-10 when compared to the baseline, although previous studies showed lower levels of IFN-g among the elderly, regardless of DRS status [20,32]. The present study shows a reduction of the microbial load of the denture and improvement in the inflammation, which may have affected the level of IL-10 and consequently of IFNγ [21].

Whelton et al. [33] highlighted that observational studies have demonstrated graded associations between higher systolic blood pressure (SBP) and diastolic blood pressure (DBP) and an increased CVD risk [34,35]. In a meta-analysis of 61 prospective studies, the risk of CVD increased in a log-linear fashion from SBP levels <115 mm Hg to >180 mm Hg and from DBP levels <75 mm Hg to >105 mm Hg [34]. In that analysis, 20 mm Hg higher SBP and 10 mm Hg higher DBP were each associated with a doubling in the risk of death from stroke, heart disease, or other vascular disease [34]. Therefore, the slight but significant reduction of 6 mmHg in SAP and 5 mmHg in MAP observed in the current study may offer benefits to elderly patients if these changes in arterial pressure are maintained over a long period of time.

Limitations of this observational study included limited follow-up and a reduced sample of patients, which may influence the inference of data for other populations. Another limitation was not using a specific questionnaire to evaluate perceived stress during contact with the patients, in order to ensure that anxiety about medical and dental care does not have an undesirable influence. In future studies, assessing other factors related to cardiovascular risks is still pertinent, these factors include total cholesterol levels, HDL, LDL, VLDL, and triglycerides.

## 4. Materials and Methods

### 4.1. Patient Selection

This manuscript reports results from a controlled observational study about the effect of the treatment of denture-related stomatitis, for 10 days, on local inflammatory factors and hemodynamic responses to arterial pressure. The variables were measured at the beginning of the study (baseline) and after 10 days of the current treatment. The ethics committee at the School of Dentistry of Ribeirão Preto, University of Sao Paulo approved the trial protocol (CAAE: 93712418.1.0000.5419).

In the first dental visit, the patients were informed about the nature of the study by three researchers (C.B.A., C.V.F., and A.B.R.^1^). After agreeing to participate, they were evaluated according to the inclusion and exclusion criteria. Individuals who met the eligibility criteria were presented with the Free and Informed Consent Term for acknowledgment and signature. After that, the participants were evaluated through a clinical-anamnestic examination regarding sociodemographic aspects and the general health status and condition of their oral cavity. Participants were edentulous patients who requested treatment at the clinics of the School of Dentistry of Ribeirão Preto, where the study took place, being enrolled from August 2019 to February 2020.

The inclusion criteria were individuals from both genders, with good general health, defined by the absence of conditions that could alter the local inflammatory process and oral microbiota: for example, diseases such as uncontrolled diabetes mellitus not accompanied by doctors, severe cardiovascular disorders involving the use of a pacemaker, stroke, myocardial infarction in the last 12 months, drug addiction, radiation therapy in the head and neck region, and a history of chemotherapy for neoplasms. As well as these criteria, we also included complete edentulous patients, users of upper and/or lower conventional complete dentures in acrylic resin, with denture stomatitis types IA, IB, II, or III, according to the Newton Modified Classification [34]. Exclusion criteria included any oral mucosal lesion apart from DRS, such as hyperplasias, papillomas, or thrush. As well as this, we also excluded systemic or local conditions that are predisposed to *Candida* spp. infection, such as conditions of immunosuppression, acquired immune deficiency syndrome (AIDS), cancer, use of medications such as antibiotics, or steroidal or antifungal agents in the 3 months before the study or during follow-up; and the use of palatal brushing or disinfectant solutions as part of routine oral hygiene, as these could interfere with the implementation of the hygiene protocol that included these procedures.

### 4.2. Hygiene Protocol

Participants were instructed to brush their hard palate for 2 min with a toothbrush with soft bristles and water (CS 5460; Curaprox, Curaden Swiss, Kriens, Switzerland); and also brush their dentures with a specific denture brush (BDC 152/153; Curaprox, Curaden Swiss, Kriens, Switzerland) and neutral liquid soap for 2 min (Pleasant; Perol Commercial and Industrial Ltd., Ribeirão Preto, Brazil) 3 times a day. After dinner, the dentures were soaked in 150 mL of 0.25% sodium hypochlorite (Super Candida^®^ Indústria Anhembi, Osasco, São Paulo, Brazil) for 20 min. The dentures were to remain immersed in clean water overnight. Participants were blinded to the disinfecting agent. The neutral liquid soap and solution were placed in a dosing bottle.

### 4.3. Study Outcomes

#### 4.3.1. Denture-Related Stomatitis Remission

Patients were clinically examined and selected from a diagnosis of denture-related stomatitis degree Type IA or higher, according to the modified Newton Classification [36]: Type IA, petechial on the palatal mucosa, usually around the ducts of the palatine salivary glands; Type IB, the localized inflamed area under the denture base; Type II, generalized area of inflammation under the denture base; and Type III, papillary palatal hyperplasia associated with inflammation under the denture base. Standardized photographs of the palate were obtained (EOS Digital Rebel EF-S 18-55 with Canon MR-14 EX flash; Canon Inc) with the focus centralized on the median raphe region [12,22]. The images were transferred to the computer (C.B.A. and C.V.F.), and two researchers (C.H.S-L. and H.F.O.P.), who had previously calibrated in relation to the interpretation of the degrees of inflammation,, assigned the scores, thus confirming the degree of stomatitis.

#### 4.3.2. Biofilm Reduction and Antimicrobial Activity

For biofilm reduction analyses, the intaglio surface of the maxillary complete denture was dyed with 1% neutral red (IMBRALAB Ltd., São Paulo, Brazil), photographed (EOS Digital Rebel EF-S 18-55 with Canon MR-14 EX flash; Canon Inc, Tóquio, Japão) [11,12], and the photographs were transferred to a computer to calculate the percentage of biofilm with the aid of the NIS-Elements BR software (Nikon Instruments Inc., 2019, Tóquio, Japão). The percentage of biofilm was calculated by the relation between the number of pixels in the dyed area multiplied by 100 and the number of pixels on the intaglio denture surface [12].

For analyses of microbial load, the denture was removed from the patient′s mouth and transferred to the laboratory for biofilm collection in an aseptic zone. The dentures were placed in Petri dishes and the biofilm was collected by adsorption using a sterilized toothbrush and 10 mL of phosphate-buffered saline (PBS). The suspension was transferred to a tube containing glass beads, vortexed for 1 min, diluted from 10^0^ to 10^−3^, and seeded in Petri dishes [37] with specific culture mediums such as CHROMagar™ *Candida* (Becton Dickinson, Paris, France) for isolation of *Candida* spp.; MacConkey Agar (Himedia Laboratories PVI Ltd., Mumbai, India) for isolation of Gram-negative microorganisms; Manitol Agar Salt (Kasvi Imp. e Dist. de Prod. para Laboratórios Ltd., Curitiba, Brazil) for isolation of *Staphylococcus* spp.; and SB20 Agar Modified by casitone for isolation of *Streptococcus* spp. The plates were incubated in a stove (De Leo Equipamentos Laboratoriais, Porto Alegre, RS, Brazil) at 37 °C for 48 h. The plates with *S. mutans* were incubated in a microaerobic environment in an anaerobiosis jar (Permution, Curitiba, PR, Brazil). Subsequently, the dentures were brushed thoroughly and returned to the participants without biofilm. To obtain a palatal microbial load, a sterile cytology brush was rubbed for 1 min on the hard palate. The active tip was stored in a tube containing glass beads and was processed in the same way as the specimens collected from the dentures [12,22]. The microbial load was determined by considering that the dilution of CFUs ranged from 0 to 300 colonies and according to the formula CFU/mL = number of colonies × 10n/q, where n is the absolute value of dilution (0, 1, 2, or 3) and q is the quantity (mL) pipetted for each dilution at inoculation (0.05 mL).

#### 4.3.3. Salivary Parameters, Identification, and Quantification of MUC 1

Saliva samples were collected from 9 to 11 am for 10 min by the method of spitting. The volume was recorded to calculate the salivary flow and the pH was measured with the aid of a pHmeter (PHTEK, Curitiba, Paraná, Brazil) [15,38]. Subsequently, saliva samples were centrifuged at 10,000× *g* for 15 min at 4 °C to remove cellular debris, and an aliquoted supernatant was stored at −80 °C for analysis by ELISA and Cytometry. For identification and quantification of MUC 1 expression (mU/mL), pure saliva samples were thawed at room temperature and measured for MUC 1 concentrations using an enzyme immunoassay kit (Enzyme-Linked Immunosorbent Assay-ELISA-RAB0375) according to the manufacturer′s instructions (Sigma-Aldrich; St. Louis, MO, USA). Previously, prepared curve standards and samples had been pipetted into 96-well plates sensitized with the capture antibody. After two and a half hours, the plates were washed and dried. A biotinylated detection antibody was added, and the plates were incubated again for one hour. The solution was discarded, and washings were performed. After the plates were dry, the streptoavidin-hydrogen peroxide conjugate was added. After forty-five minutes, the solutions were discarded again, and the plate wells were washed, dried, and tetramethylbenzidine (TMB) was added and incubated for thirty minutes in the dark. The assay process finished with the addition of stop solution, and plate readings at 405 nm were measured on an ELISA reader (Thermo ScientificTM MultiskanTM go Microplate Spectrophotometer, Waltham, MA, USA). As a control, wells without saliva were added only with the reagents present in the Kit. The assay was performed in duplicate. According to the absorbance curve, the results were presented as the mean difference between the readings in optical density units (OD) in experimental and control wells.

#### 4.3.4. Evaluation of Cytokines

Salivary concentrations of IL-6, TNFα, IL-4, IL-2, IL-17, IFNγ, and IL-10 were measured using a BD™ Cytometric Bead Array (CBA) Human Th1/Th2/Th17 (RRID: AB_2869353) Cytokine (BD; San Jose, CA, USA) in a single duplicate sample. Quantification was performed following the manufacturer’s instructions. Briefly, seven populations of beads with different fluorescence intensities were conjugated with a capture antibody specific for each cytokine, mixed to form the CBA. Bead populations were visualized according to their respective fluorescence intensities: from least to most brilliant. In CBA, cytokine capture beads are mixed with a detection antibody conjugated with fluorochrome (PE) and then incubated with the samples. Tubes for acquisition were prepared with the sample, bead mix, and detection reagent. The same procedure was performed to obtain the standard curve. The tubes were homogenized and incubated for three hours at room temperature in the dark. The results were generated in graphs and tables.

#### 4.3.5. Evaluation of Arterial Pressure

Arterial pressures were recorded at the baseline and after the treatment of DRS. The participant’s arterial pressure was indirectly measured by the oscillatory sphygmomanometer method using an automated device (HEM7130, Omron Healthcare Brazil, São Paulo, Brazil); three measurements were performed at 5 min intervals recording the systolic and diastolic arterial pressure.

### 4.4. Statistical Analysis

The paired Wilcoxon and Fisher Tests evaluated biofilm removal and the CFU of microorganisms. The paired T test was used to assess salivary parameters (flow and pH), salivary MUC 1, cytokines, and arterial pressure before and after the use of the current hygiene protocol. All tests were conducted using the SPSS 25.0 statistical software (SPSS Inc., Chicago, IL, USA) with a significance level of 5%.

## 5. Conclusions

The current hygiene protocol reduced the score of the DRS, the biofilm percentage of the inner surface, and microbial load count of the *Candida* spp. on dentures. In addition, the protocol promoted improvement in local inflammatory factors with an increase in MUC 1 and a decrease in IL-6, Il-2, Il-10, and INFγ combined with a reduction in systolic and media arterial pressures.

## Figures and Tables

**Table 1:** Sociodemographic data of the sample expressed in absolute numbers (percentage).

Variable	Total
Gender	
Female	26 (78.79%)
Male	7 (21.21%)
Marital status	
Married	14 (42.42%)
Unmarried	4 (12.12%)
Divorced	5 (15.15%)
Widowed	6 (18.18%)
Separated	3 (9.09%)
Other	1 (3.03%)
Lives with	
Alone	8 (24.24%)
Family	24 (72.73%)
Friend	1 (3.03%)
Education	
Illiterate	4 (12.12%)
High school or less	28 (30.3%)
Higher education	1 (57.58%)
Income	
No income	0
1 to 3 salaries	31 (93.94%)
4 to 7 salaries	1 (3.03%)
7 to 10 salaries	1 (3.03%)

**Table 2:** Comparison of denture-related stomatitis score at the baseline and after treatment with the hygiene protocol; (95% CI).

	Baseline	Treatment	*p* **
Mean (SD)	2.77 (0.80)	1.73 (1.09)	<0.001
Median (CI)	3.00 (2.00; 3.00)	2.00 (1.00; 3.00)	

** Wilcoxon test (*p* < 0.05). SD: standard deviation; CI: confidence interval.

**Table 3:** Comparison of biofilm percentage on inner surface of the upper denture at the baseline and after treatment (95% CI).

	Baseline	Treatment	*p* **
Mean (SD)	26.29 (16.66)	5.10 (8.45)	<0.001
Median (CI)	23.22 (15.18; 38.03)	1.28 (0.22; 6.27)	

** Wilcoxon test (*p* < 0.05). SD: standard deviation; CI: confidence interval.

**Table 4:** CFU counts of *Candida* spp., Gram-negative bacteria, *Staphylococcus* spp., and *S. mutans* at the baseline (B) and after treatment (T).

		Denture			Palate		
		B	T	*p* **	B	T	*p* **
*Candida* spp.	Mean	3.64	1.20	<0.001	0.72	0.62 (1.28)	0.798
	(SD)	(2.26)	(1.76)		(1.08)		
	Median	4.54	0.0		0.0	0.0	
	(CI)	(1.79; 5.41)	(0.0; 2.48)		(0.0; 1.61)	(0.0; 0.0)	
Gram-negative	Mean	1.31	0.52	0.004	0.64	0.93	0.259
	(SD)	(1.26)	(1.20)		(1.21)	(1.32)	
	Median	1.32	0.0		0.0	0.0	
	(CI)	(0.0; 2.0)	(0.0; 0.0)		(0.0; 132)	(0.0; 1.61)	
*Staphylococcus* spp.	Mean	1.10	0.27	0.001	1.05	0.82	0.798
	(SD)	(0.96)	(0.63)		(0.95)	(0.90)	
	Median	1.32	0.0		1.32	0.0	
	(CI)	(0.0; 1.91)	(0.0; 0.0)		(0.0; 1.61)	(0.0; 1.79)	
*S. mutans*	Mean	3.9	1.05	<0.001	2.69	1.32	<0.001
	(SD)	(2.7)	(2.07)		(1.52)	(0.88)	
	Median	4.34	0.0		3.0	1.0	
	(CI)	(1.32; 6.34)	(0.0; 1.32)		(1.0; 3.99)	(1.0; 1.0)	

** Wilcoxon test (*p* < 0.05). SD: standard deviation; CI: confidence interval; B: baseline; T: treatment.

## Data Availability

Not applicable.
